# Granular Cell Tumors of the Musculoskeletal System and Peripheral Nerves: A Systematic Review of Clinical Presentations, Treatments, and Outcomes

**DOI:** 10.3390/diagnostics16060885

**Published:** 2026-03-17

**Authors:** Edoardo Ipponi, Antonio D’Arienzo, Francesco Rosario Campo, Fabrizia Gentili, Fabio Cosseddu, Lorenzo Andreani, Paolo Domenico Parchi

**Affiliations:** Department of Ortohpedics and Trauma Surgery, University of Pisa, Via Paradisa 2, 56124 Pisa, Italy

**Keywords:** soft tissue tumor, symptoms, pain, swelling, recurrence, metastasis, survival

## Abstract

**Background:** Granular cell tumors (GCTs) are rare neoplasms that may also involve the musculoskeletal system and peripheral nerves of the extremities. In these locations, their clinical presentation, management, and outcomes remain poorly characterized. **Methods:** A systematic review was conducted according to PRISMA guidelines. PubMed, MEDLINE, EMBASE, and Scopus were searched for articles published between 1975 and 2025 reporting GCTs arising from the musculoskeletal system or peripheral nerves, with available data on clinical presentation and treatment. Tumor location and size, symptoms, treatment modality, and oncological outcomes (recurrence or metastasis) at the latest follow-up were extracted. **Results:** Forty articles describing 67 cases were included (50 females, 17 males). Tumors showed benign (47) or atypical (2) behavior in 49 cases and malignant features in 18 cases. The mean largest tumor diameter was 44 mm, and malignant lesions were significantly larger than benign ones. Thirty-one lesions were located in the lower limbs, 25 in the upper limbs, and 11 had central musculoskeletal localizations. Swelling was the most common presenting symptom (92%), followed by pain (40%). Surgical excision was performed in all patients except one, who underwent primary amputation. Adjuvant chemotherapy or radiotherapy was sporadically used in malignant cases (two cases each). Among the malignant cases with reported oncological follow-up, 44% developed distant metastases, and one (5.6%) also experienced local recurrence. Only one benign GCT recurred (2%), whereas all other non-malignant lesions remained CDF (98%). **Conclusions:** Although rare, GCTs should be considered in the differential diagnosis of musculoskeletal soft-tissue tumors, given their potential for malignant behavior and metastatic spread.

## 1. Introduction

Granular cell tumor (GCT), also known as Abrikossoff tumor, is an uncommon soft tissue neoplasm composed of polygonal to occasionally spindle-shaped cells with abundant eosinophilic, finely granular cytoplasm [1]. Historically, the histogenesis of GCT has been debated for nearly a century. Abrikossoff first described the lesion in 1926 and proposed a myogenic origin, which led to the early designation of granular cell myoblastoma [2]. With the later development of immunohistochemistry and electron microscopy, this hypothesis was progressively abandoned in favor of a Schwann cell lineage, supported by consistent expression of neural crest-associated markers and ultrastructural evidence of lysosome-rich cytoplasmic granules [3]. Recognizing GCT as a nerve sheath tumor also explains its broad anatomic distribution and its origin from different superficial and deep tissues [1]. In the 2020 World Health Organization (WHO) classification of soft tissue tumors, granular cell tumor is categorized among peripheral nerve sheath tumors, following its Schwannian differentiation [4].

From an epidemiologic standpoint, granular cell tumors are rare. In soft-tissue pathology series, GCT is frequently cited as representing approximately 0.5% of all soft-tissue tumors, with a female predominance and a peak incidence in middle adulthood. However, to our knowledge, large-scale dedicated epidemiological studies are not available [5]. The anatomic distribution is wide. Many reviews described the head and neck region as the most frequent site of origin [1,6]. Other common locations include skin and subcutaneous tissue, the gastrointestinal tract, and the breast, reflecting the tumor’s tendency to arise in tissues rich in peripheral nerve fibers [1,5,6]. Despite its widespread anatomical distribution, GCTs arising from the musculoskeletal apparatus or directly from major peripheral nerves are particularly uncommon [7]. Literature on this topic has therefore been generally limited to isolated case reports and small institutional experiences [7].

Clinically, GCTs are frequently described as slow-growing, firm nodules that may remain asymptomatic for prolonged periods [1]. In musculoskeletal presentations, symptoms reported in the literature commonly include a palpable mass or localized swelling [7]. These symptoms often depend on the depth, size, and mechanical impingement of the nodular mass on nearby tissues. When the tumor develops within or adjacent to a peripheral nerve, patients may also present with paresthesia, neuropathic pain, or motor deficits, eventually mimicking more common peripheral nerve sheath tumors such as schwannoma or neurofibroma [4]. Still, little is known about the clinical characteristics and pathways of presentation for musculoskeletal GCTs.

Despite the importance of clinical presentation in orienting physicians and initiating the diagnostic pathway, radiologic evaluation remains central to characterizing these lesions. Imaging is a reliable tool for assessing neoplasms’ characteristics and behavior and for guiding biopsy and surgical planning. Ultrasound can assist in evaluating superficial masses and guide eventual biopsies, whereas magnetic resonance imaging (MRI) is preferred for deep lesions. In intramuscular granular cell tumors of the extremities, MRI has been reported to show signal intensity isointense to skeletal muscle on T1-weighted sequences, heterogeneous signal on T2-weighted sequences slightly higher than muscle but lower than fat, and contrast enhancement [7]. A peripheral rim of high signal on T2-weighted images has also been described in intramuscular lesions [7]. While such patterns may raise suspicion, overlap with other soft tissue tumors is substantial, and imaging must be interpreted in the broader clinical context [8].

Histopathologic evaluation is mandatory to establish a definitive diagnosis of GCT. Classic GCT is composed of polygonal cells with abundant granular eosinophilic cytoplasm [1]. The cytoplasmic granules are typically periodic acid–Schiff (PAS)-positive and diastase-resistant, consistent with their lysosomal content [1]. Immunohistochemistry supports Schwannian differentiation, with most conventional tumors expressing S-100 and SOX10 and frequently coexpressing CD68 and other markers [3]. GCTs can present a spectrum of biological behaviors. Fanburg-Smith et al. proposed widely used histologic criteria to stratify tumors as benign, atypical, or malignant, based on six features (including necrosis, spindling, and increased mitotic activity) [9]. Subsequent work has further discussed and tested these diagnostic criteria in cohorts of malignant granular cell tumors [10]. Although the vast majority of GCTs located throughout the human body are benign, malignant behavior has been reported in a small minority of cases [5,9].

Once the diagnosis has been established, appropriate treatment should be initiated to achieve local control and disease eradication. Although there are no universally accepted location-specific guidelines for managing GCT across all anatomic sites, surgery remains the cornerstone of treatment [1]. For benign lesions, complete excision is generally pursued to reduce the risk of local recurrence, particularly when margins are involved [5]. For malignant GCT, wider resections with clear margins and vigilant surveillance are typically advocated due to the risk of local recurrence and distant metastasis [9,10,11]. The roles and efficacy of radiotherapy and systemic chemotherapy remain debated, as their use is limited to case-based evidence [11]. Importantly, recurrence risk may vary across anatomic sites and clinical contexts, and some series (e.g., breast GCT) have reported very low recurrence even with close or positive margins [12].

Because granular cell tumors are uncommon overall—and distinctly rare in the musculoskeletal system and peripheral nerves—the literature remains fragmented and heterogeneous, with much of the published experience derived from case reports and small series [7]. As a result, the true spectrum of clinical presentation, including the relative frequency of pain and other symptoms, remains incompletely characterized. Similarly, the oncologic and functional outcomes after treatment remain scarcely documented [7].

This systematic review aims to synthesize the available evidence on musculoskeletal and peripheral nerve granular cell tumors, with a specific focus on clinical presentations, treatments, and outcomes, to summarize current knowledge and identify persistent gaps warranting future investigation.

## 2. Materials and Methods

A systematic review of the literature was conducted in accordance with the Preferred Reporting Items for Systematic Reviews and Meta-Analyses (PRISMA) guidelines, using the PRISMA checklist and flow diagram [13] (Supplementary Materials). The systematic review has been registered online using the INPLASY portal (registration number INPLASY202620034).

### 2.1. Search Strategy, Inclusion and Exclusion Criteria

A comprehensive search of the PubMed, MEDLINE, EMBASE, and Scopus databases using the following keywords included in this search line: *(granular cell tumor) AND ((musculoskeletal) OR (Limb) OR (Shoulder) OR (Arm) OR (Elbow) OR (Forearm) OR (Wrist) OR (Hand) OR (Hip) OR (Thigh) OR (Knee) OR (Leg) OR (Ankle) OR (Foot) OR (Muscle) OR (Articulation))*. To our knowledge, no MeSH/Emtree terms were intentionally used for our research. All papers published between 1975 and 2025, available as of 1st November 2025, the day of data extraction, were included in our research. All original articles reporting on patients diagnosed with granular cell tumors arising from peripheral nerves, involving the musculoskeletal system, and located in the upper or lower limbs that required surgical management were included. The literature search was independently conducted by three investigators (E.I., A.D., F.R.C.). Eligibility was restricted to studies published in peer-reviewed journals. Each investigator independently screened titles and abstracts, followed by a comprehensive full-text assessment of all eligible articles. Data extraction was performed independently by the reviewers to minimize the risk of selection bias and data extraction errors.

Inclusion criteria were (1) a confirmed histological diagnosis of granular cell tumor, (2) a surgical treatment aimed to eradicate the neoplasm, and (3) data regarding the clinical presentation of the disease or the oncological outcomes of treated patients. Exclusion criteria were (1) articles that did not mention or provide data on the surgical treatment, (2) articles that did not report on either patients’ pre-operative clinical presentation or their postoperative outcome, (3) pre-clinical studies, (4) literature reviews without any new cases, and (5) papers written in languages other than English. During the review, authors also excluded (6) articles reporting on cases with diagnoses different than GCT, such as schwannoma or neurofibroma, and (7) cases whose lesions were localized solely in the skin or superficial subcutis so that neither the anatomical picture, the clinical presentation, nor the surgical treatment involved the musculoskeletal system. Conversely, cases in which the lesion or surgical treatment involved the muscular fascia or deeper layers were included.

**Figure 1:**
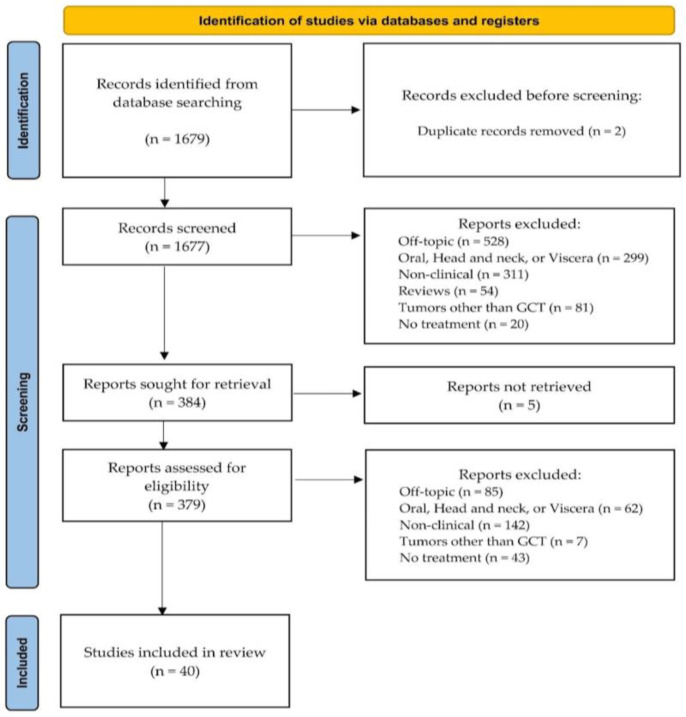
A schematic representation of our study’s PRISMA flow-chart.

All articles were initially screened for relevance based on their titles and abstracts. The full text was obtained if the abstract did not allow the investigators to assess inclusion and exclusion criteria. Given the limited availability of large case series and the generally low level of evidence among the existing literature, studies ranging from Level I to Level V evidence were included in the present analysis. Detailed case reports were also included. The study selection process, conducted in accordance with the PRISMA guidelines [13], is summarized in Figure 1.

### 2.2. Data Collection for Review Purpose

For each included article, the year of publication and study design were recorded, distinguishing between case reports and case series. The number of patients, along with their age and sex, was extracted from each study. For each evaluated lesion, the anatomical location and lesion size were also documented. When mentioned, symptoms were also documented. In particular, the incidence of pain and local swelling was reported in detail. Malignancy was reported primarily when explicitly reported in the original studies. When reported by the authors, the tumor classification according to the Fanburg-Smith criteria [9] was recorded. When lesions were not explicitly classified as benign, atypical, or malignant, they were categorized based on the available histopathologic features according to the Fanburg-Smith criteria, including cytologic atypia, mitotic activity, necrosis, spindling, prominent nucleoli, and nuclear pleomorphism. Clinical behavior was also considered; in particular, cases with documented metastatic spread were classified as malignant when not otherwise specified. The surgical treatment of choice was recorded, along with any neoadjuvant or adjuvant pharmacological or radiation therapies. The follow-up for each case report and the mean follow-up across all case series were noted. Percentages and rates for the individual items were calculated from the articles reporting on them. We evaluated whether patients experienced local recurrence and the time interval between treatment and the diagnosis of recurrence. Metastatic lesions were also recorded.

### 2.3. Quality Assessment

The Joanna Briggs Institute (JBI) Critical Appraisal tools were used to assess heterogeneity in study design and methodology across the selected cohort studies, case series, and case reports to evaluate their quality for inclusion in this systematic review. Each item on the checklist is rated with one of four possible responses: “yes”, “no”, “unclear”, or “not applicable” [14]. A dedicated bias assessment for randomized studies was not performed because there were none.

### 2.4. Statistical Analysis

Statistical analyses were conducted using Stata SE version 13.1 (StataCorp LLC, College Station, TX, USA). The rates of complications and local recurrences were either recorded or calculated for each study included in the analysis. Due to the limited sample sizes, the heterogeneity among studies, and their retrospective designs, a meta-analysis was not performed. The rarity of these tumors and the limited number of publications on this topic required the inclusion of studies with varying designs, resulting in considerable heterogeneity in clinical presentation, tumor location and size, and therapeutic strategies. Furthermore, differences in study objectives may have affected the reported outcomes.

The absence of large cohorts or complex study designs precluded the use of Egger tests to assess publication bias. Generative artificial intelligence (GenAI) (ChatGPT 5.2, OpenAI Inc., San Francisco, CA, USA) has been used to generate graphics from our collected data and to double-check the statistical analysis.

## 3. Results

**Figure 2:**
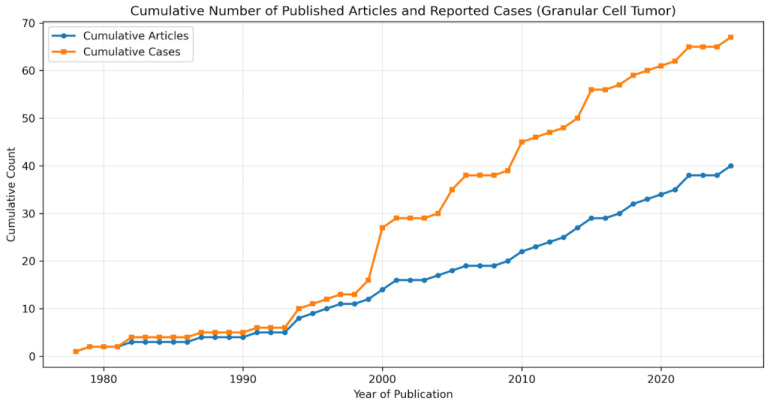
Graphic representation of the cumulative number of published articles (orange line) and reported cases (blue line) until 2025.

Forty articles met our inclusion and exclusion criteria and were included in our review [7,8,15,16,17,18,19,20,21,22,23,24,25,26,27,28,29,30,31,32,33,34,35,36,37,38,39,40,41,42,43,44,45,46,47,48,49,50,51,52]. There were eight case series and thirty-two case reports. A total of 67 cases were reported. The yearly distribution of articles and the cumulative number of cases reported in the literature over the years are shown in Figure 2.

A summary of all the case series and case reports included in our review is available in Table 1.

### 3.1. Quality Assessment

Due to size limitations, none of the included case series provided statistical analyses. Therefore, Q10 was designed to be unavailable in all of them. Four of the 8 case series articles included in our review answered yes to all remaining queries in the JBI checklist. The remaining four articles were found to be unclear in at least one of the areas investigated by the checklist but were still considered worthy of being maintained in our review. The JBI quality assessment of all the included case series was reported in detail in Table 2.

The JBI quality assessment was also conducted for all included case reports. Twenty-one of the 32 case report articles included in our review complied with all eight queries in the JBI checklist. The remaining eleven case reports were considered worthy of inclusion in our review, despite being unclear or unsatisfactory on at least one query. The JBI quality assessment of all the included case reports was reported in detail in Table 3.

### 3.2. Demographics

**Figure 3:**
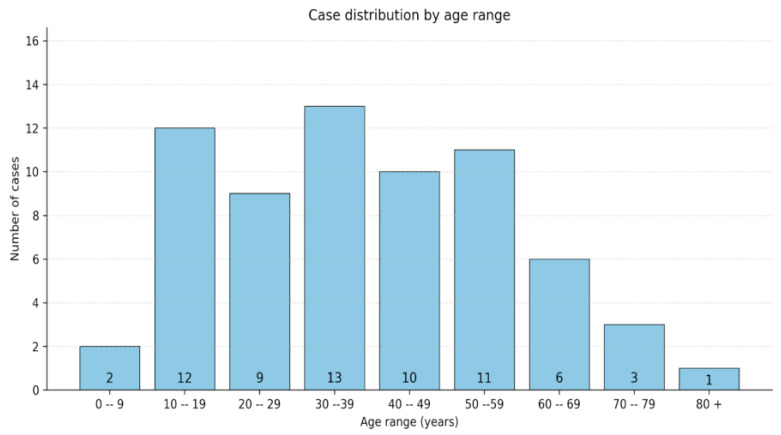
A graphic distribution of patients, sorted by their age groups (decades).

Among the 67 cases included in our review, 50 (78.6%) were females, and 17 (21.4%) were males. According to a unilateral bimodal test, females were significantly more frequent in our study group compared to males (*p* < 0.0001). The mean age at diagnosis was 38.4 (10–81). No significant predilection of any decade of life emerged from our review [7,8,15,16,17,18,19,20,21,22,23,24,25,26,27,28,29,30,31,32,33,34,35,36,37,38,39,40,41,42,43,44,45,46,47,48,49,50,51,52]. The age distribution is pictured in Figure 3.

### 3.3. Anatomical Distribution

**Figure 4:**
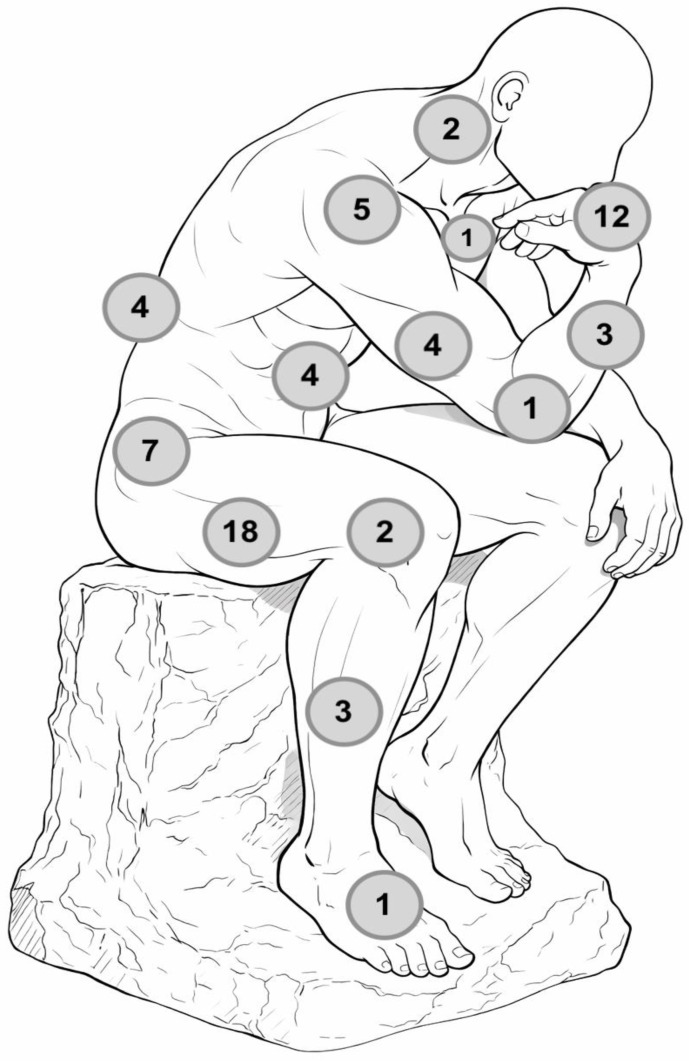
Figurative distribution of granular cell tumors included in our literature review. The number in each circle indicates the number of cases in the area where the circle is located.

The anatomical locations of the tumors were documented for all cases included in our review. Most granular cell tumors (GCTs) originated in the limbs. Of the 67 lesions analyzed, 31 cases (46.3%) were in the lower limbs. Within this category, the thigh was the most frequently affected area, with 18 cases (26.9% of all cases), followed by the pelvic girdle with 7 cases (10.4%). Other parts of the lower limb included the knee (2 cases; 3.0%), the leg (3 cases; 4.5%), and the foot (1 case; 1.5%), which were less commonly involved. In the upper limb, there were 25 GCTs (37.3%). Nearly half of these tumors were found in the hands (12 cases; 17.9%), while the rest were in the scapular girdle (5 cases; 7.5%), the arms (4 cases; 6.0%), the elbows (1 case; 1.5%), and the forearms (3 cases; 4.5%). Additionally, 11 GCTs (16.4%) arose from areas in the central body: 4 from the abdominal wall (6.0%), 4 from the spine and dorsum (6.0%), 2 from the neck (3.0%), and 1 from the chest (1.5%) [7,8,15,16,17,18,19,20,21,22,23,24,25,26,27,28,29,30,31,32,33,34,35,36,37,38,39,40,41,42,43,44,45,46,47,48,49,50,51,52]. The distribution of granular cell tumors in our study is graphically summarized in Figure 4.

### 3.4. Symptoms: Pain and Swelling

Data on the clinical presentation of granular cell tumors were reported in 39 articles, covering 66 patients [7,8,15,16,17,18,19,20,21,23,24,25,26,27,28,29,30,31,32,33,34,35,36,37,38,39,40,41,42,43,44,45,46,47,48,49,50,51,52]. Only one case (1.5%) had an incidental diagnosis, presenting to medical attention without any signs or symptoms [22]. Swelling was the most common symptom, being documented in 61 out of 66 cases (92.4%). Twenty-seventeen patients (40.1%) experienced local pain, whereas 39 (59.1%) did not. No difference in pain rates was found among lesions localized to the upper limbs, lower limbs, or central body. Irradiation of pain or dysesthesia distal to the lesion was reported by three patients (7.7%).

### 3.5. Lesion’s Size

**Figure 5:**
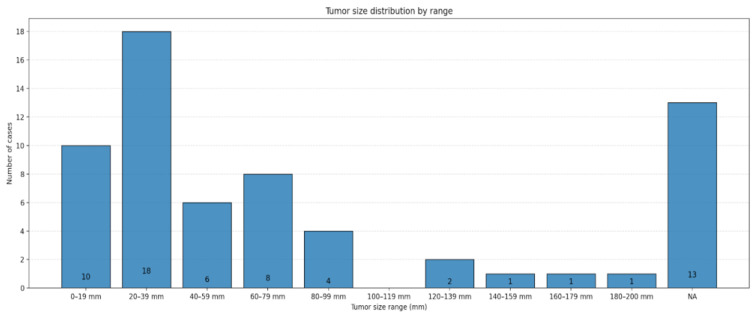
A graphic distribution of studied lesions, sorted by their larger diameter (in mm). NA = Not available.

Lesion size was reported in 36 articles, which together covered 51 cases [7,8,16,17,18,19,20,21,22,23,24,25,26,28,29,30,31,32,33,34,35,36,37,38,39,40,41,42,43,44,45,46,47,48,49,52]. The mean lesion diameter was 4.4 cm (0.5–20.0). In total, 62.7% of all lesions had diameters between 2 and 8 cm, and only 10% were larger than 10 cm. The size distribution of the included cases is schematically shown in Figure 5.

### 3.6. Benign, Atypical, and Malignant GCTs

**Figure 6:**
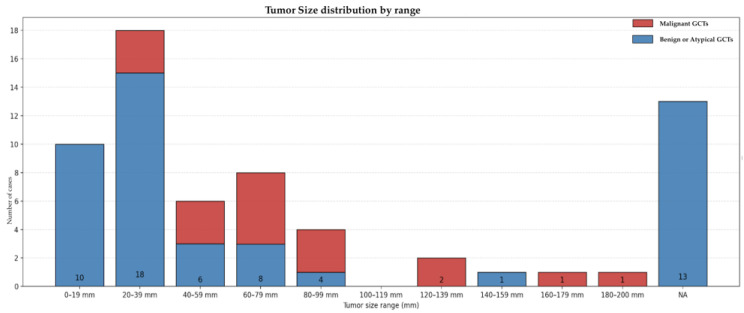
Tumor size distribution by range, dividing benign (blue) and malignant (red) GCTs.

A total of 18 cases (26.9%) were classified as malignant GCTs [7,8,18,22,25,26,28,29,33,37,39,42,43,44,48,52]. Patients with malignant lesions included 13 females (72.2%) and 5 males (27.8%), with a mean age of 51.3 years. Lesions arose from the upper limb in three cases, the lower limb in ten, the neck and back in two cases each, and the abdominal wall in one. The mean lesion size was 8.1 cm (range 2–20 cm) (Figure 6).

The authors reported two cases as atypical GCTs. The remaining 47 cases (74.6%) were reported as benign and did not meet any of the Fanburg-Smith criteria [7,8,15,16,17,18,19,20,21,23,27,30,31,32,34,35,36,38,40,41,45,46,47,49,50,51].

According to a two-tailed Student’s *t*-test, malignant lesions were significantly larger than the others (*p* = 0.001).

### 3.7. Treatments

All patients in our study underwent surgical treatment, aiming for neoplasm resection. All limb lesions were treated with limb-sparing surgery, and no amputations were performed [7,8,15,16,17,18,19,20,21,22,23,24,25,26,27,28,29,30,31,32,33,34,35,36,37,38,39,40,41,42,43,44,45,46,47,48,49,50,51,52]. Information on the quality of margins was provided for 28 patients. Among them, 26 had wide or marginal resections (R0 or R1), while the remaining two cases were treated with intralesional approaches (R2). In one case, the loss of muscle and fascia in the abdominal wall necessitated the implantation of polypropylene mesh [21]. In two cases, the resection resulted in loss of skin coverage, requiring skin grafts or skin flaps [30,35].

Four patients with malignant GCTs received adjuvant chemotherapy [7,25,42], whereas two cases received local adjuvant radiotherapy [7,44].

A summary of reported treatments is provided in Table 4.

### 3.8. Post-Operative Follow-Up and Outcomes

The post-operative follow-up was exhaustively described in 60 of 67 cases [7,8,15,23,25,26,28,29,30,31,32,33,35,36,37,38,39,40,41,42,44,45,47,48,49,50,51,52]. Among them, 45 had benign (43) or atypical (2) GCTs, while 15 had malignant lesions [7,8,18,22,25,26,28,29,33,37,39,42,43,44,48,52]. Their mean post-operative follow-up was 26.1 months.

Only one of 43 benign neoplasms (2.2%), localized in the hand, had a local recurrence 11 months after surgery [19]. None of the two atypical tumors had local recurrences. The recurrence rate of malignant GCTs treated with marginal or wide resections was 7.1%, with one of 14 cases experiencing local recurrence 3 months after surgery [7].

Metastases were reported in 8 of 15 cases with malignant GCTs (53.3%) [7,8,18,22,25,29,37,42]. Lungs were the most common site of secondary lesions, involved in seven of the eight patients (46.7%). Lymph nodes’ involvement was documented in three cases (20%), splanchnic abdominal involvement in one case (liver; 6.7%), and skeletal localization in another case (spine and pelvis; 6.7%).

At their latest follow-up, of the 15 cases with malignant tumors with detailed follow-up, six died of disease (DOD) or of other causes (DOC) (40%). Two were alive with disease (AWD) (13.3%), while the remaining seven cases were continuously disease free through their post-operative intercourse (CDF) (46.7%) [7,8,15,23,25,26,28,29,30,31,32,33,35,36,37,38,39,40,41,42,44,45,47,48,49,50,51,52].

## 4. Discussion

Granular cell tumors are a group of uncommon, heterogeneous neoplasms whose behavior within the musculoskeletal system and peripheral nerves is only partially characterized because of their rarity [1,2,3,4]. While GCTs are well known in head and neck oncology, their presentation in deep soft tissues, muscles, and major peripheral nerves of the extremities is distinctly infrequent [5,6]. As a result, much of the existing knowledge about GCTs comes from isolated case reports or very small series, which often focus on specific anatomical locations or atypical clinical courses. The present systematic review was therefore conceived to integrate these fragmented observations and provide a more comprehensive overview of musculoskeletal and peripheral nerve GCTs, with particular attention to clinical presentation, treatment strategies, and their oncological outcomes [1,6,7,8,15,16,17,18,19,20,21,22,23,24,25,26,27,28,29,30,31,32,33,34,35,36,37,38,39,40,41,42,43,44,45,46,47,48,49,50,51,52]. This systematic review summarizes 67 reported cases of granular cell tumors arising from the musculoskeletal system and peripheral nerves. The tumors predominantly affected female patients and mainly involved the extremities, particularly the lower limbs and the hand. Clinically, they typically presented as slowly growing masses associated with local swelling, while pain was reported in a substantial proportion of cases. Surgical excision provided excellent local control in benign tumors, with a very low recurrence rate. By contrast, malignant granular cell tumors were associated with frequent distant metastases and disease-related mortality despite surgical treatment.

The demographic profile from our analysis [1,6,7,8,15,16,17,18,19,20,21,22,23,24,25,26,27,28,29,30,31,32,33,34,35,36,37,38,39,40,41,42,43,44,45,46,47,48,49,50,51,52] shows a clear predominance of female patients, with a female-to-male ratio of 2.9:1 [7,8,15,16,17,18,19,20,21,22,23,24,25,26,27,28,29,30,31,32,33,34,35,36,37,38,39,40,41,42,43,44,45,46,47,48,49,50,51,52]. This finding is consistent with some early suggestions in the literature. Unlike other rare musculoskeletal tumors that preferentially affect younger individuals, GCTs in our reviewed cohort were diagnosed over a broad age range, with a tendency toward middle adulthood. This broad age distribution and the absence of a sharply defined age peak suggest that diagnostic awareness of GCTs should be maintained across all ages and not limited to the typical age windows commonly associated with most primary bone or soft-tissue tumors [15,53].

From an anatomical perspective, our findings highlight a clear predominance of lesions arising in the extremities, particularly in the lower limbs. The thigh and pelvic girdle accounted for the majority of cases, followed by the upper limb, with the hand as the most frequently involved site. This distribution may be correlated with the abundance of muscular tissue and peripheral nerve structures in these regions. Central musculoskeletal localizations, including paraspinal, abdominal wall, and dorsal regions, were less frequently reported but remain clinically significant due to their diagnostic complexity and surgical implications [7,8,15,16,17,18,19,20,21,22,23,24,25,26,27,28,29,30,31,32,33,34,35,36,37,38,39,40,41,42,43,44,45,46,47,48,49,50,51,52].

Clinically, most patients presented with a slowly enlarging mass, often accompanied by local swelling. The only cases in which the mass was undetectable were deep within the body. Pain was reported in a substantial subset of cases. Soreness was not limited to deeply seated or near major neurovascular structures; it also occurred in patients with relatively peripheral or superficial lesions [7,8,15,16,17,18,19,20,21,23,24,25,26,27,28,29,30,31,32,33,34,35,36,37,38,39,40,41,42,43,44,45,46,47,48,49,50,51,52]. Compared with GCTs arising in mucosal or subcutaneous tissues, musculoskeletal tumors appear more likely to become symptomatic, likely due to mechanical interaction with surrounding muscles, tendons, or nerves [7,8,15,16,17,18,19,20,21,23,24,25,26,27,28,29,30,31,32,33,34,35,36,37,38,39,40,41,42,43,44,45,46,47,48,49,50,51,52,54]. Neurological symptoms were uncommon but noteworthy, especially in tumors originating from or closely adherent to peripheral nerves. This finding should be noted, as sensory disturbances or neuropathic pain could occasionally lead to diagnostic confusion with more common nerve sheath tumors such as schwannomas [55,56].

Reviewed cases also showed heterogeneity in tumor size, with most ranging from 2 to 8 cm. The tumor’s larger diameter emerged as a relevant prognostic parameter for distinguishing benign from malignant behavior [7,8,16,17,18,19,20,21,22,23,24,25,26,28,29,30,31,32,33,34,35,36,37,38,39,40,41,42,43,44,45,46,47,48,49,52]. Malignant GCTs were, on average, significantly larger than their benign counterparts, reinforcing previous suggestions that size may serve as a warning sign for aggressive disease [7,8,18,22,25,26,28,29,33,37,39,42,43,44,48,52]. Nevertheless, size alone cannot reliably predict malignancy, as overlap between benign and malignant lesions persists. The definitive assessment for malignancy should, in fact, rely on comprehensive histopathological evaluation—including assessment of mitotic activity, nuclear pleomorphism, necrosis, and proliferative indices—rather than on clinical or radiological features alone. The molecular genetics of granular cell tumors remains debated. Whole-genome sequencing of a single malignant GCT by Wei et al. [57] identified a loss-of-function mutation in BRD7, a tumor suppressor candidate involved in chromatin remodeling and TP53-mediated transcriptional regulation, as well as a missense mutation in GFRA2, a component of receptor tyrosine kinase signaling. Based on these findings, the authors suggested that malignant granular cell tumors may be driven by selected molecular alterations rather than high genomic complexity, with potential implications for targeted therapeutic strategies [57]. In fact, accurate diagnosis remains a central challenge in the management of soft tissue musculoskeletal tumors, including GCTs. The differential diagnosis includes a wide spectrum of benign and malignant entities, including desmoid-type fibromatosis, schwannoma, and soft-tissue sarcoma [8,37]. Misdiagnosis may lead to inappropriate surgical planning or insufficient oncological treatment. Therefore, close collaboration among orthopedic surgeons, radiologists, and pathologists is essential to ensure accurate diagnosis of GCT, assess potential malignancy, and determine optimal management [58].

The percentage of malignant GCTs included in our review was 27% [7,8,18,22,25,26,28,29,33,37,39,42,43,44,48,52]. This proportion was significantly higher than that previously reported in the broader GCT literature [7,8,15,16,17,18,19,20]. Although it may reflect reporting bias, as deeper, larger, or more aggressive tumors in the musculoskeletal system are more likely to undergo surgical resection and subsequent publication, these data should raise clinicians’ and pathologists’ awareness of GCTs and their incidence of malignancy [9,10,16,18].

Once the diagnosis of granular cell tumor has been established, an appropriate therapeutic plan should be implemented. Surgical excision was the primary treatment modality across all reported cases in our review. Limb-sparing procedures were feasible for all extremity lesions, highlighting the generally favorable anatomical resectability of these tumors despite frequent fibrous septa in the peripheral areas of the neoplasm [7,8,15,16,17,18,19,20,21,22,23,24,25,26,27,28,29,30,31,32,33,34,35,36,37,38,39,40,41,42,43,44,45,46,47,48,49,50,51,52]. For benign GCT, wide local excision with histologically clear margins is usually curative. Reoperation should be recommended if margins are positive, as positive resection margins are a recognized risk factor for local recurrence [7,8,15,16,17,18,19,20,21,23,27,30,31,32,34,35,36,38,40,41,45,46,47,49,50,51]. Conversely, in anatomically constrained locations where a wide margin would entail major functional sacrifice, some authors accept a closer margin but stress the need for careful histologic assessment and close follow-up, given the infiltrative growth pattern and poor macroscopic encapsulation of these tumors [36]. After complete excision, benign GCTs demonstrated excellent local control. In fact, among benign lesions, the recurrence rate was as low as 2.2% among 45 cases. This finding confirms that surgery alone is curative in the vast majority of benign cases, especially when performed in referral centers by specialized surgeons, to achieve adequate resection margins [7,8,15,16,17,18,19,20,21,23,27,30,31,32,34,35,36,38,40,41,45,46,47,49,50,51].

In malignant GCT, wide excision remains the cornerstone of treatment, with resections analogous to those performed for most soft-tissue sarcomas. Malignant granular cell tumors had relatively poorer oncological outcomes than benign lesions, despite surgical intervention. Although local recurrence was also relatively rare among malignant GCTs (7.1%), these tumors exhibited a markedly aggressive course. Malignancy was associated with a high incidence of distant metastases, most commonly involving the lungs, and a mortality of 40% after a mean follow-up of 26 months. These observations support the classification of malignant musculoskeletal GCTs as high-risk tumors requiring careful treatment and vigilant postoperative surveillance [7,8,18,22,25,26,28,29,33,37,39,42,43,44,48,52].

The role of adjuvant therapies remains ill-defined. Radiotherapy and chemotherapy were employed sporadically and exclusively in malignant cases, with no consistent evidence of benefit. The small number of treated patients and the heterogeneity of regimens preclude definitive conclusions from the limited data available at the moment [7,25,42,44]. Consequently, therapeutic decisions in these cases should be individualized and discussed within a multidisciplinary tumor board, balancing potential benefits against treatment-related complications [7,25,36,38,42,44].

We acknowledge that our study has some limitations. The rarity of granular cell tumors has limited the number of published case series and the size of available cohorts. These conditions impeded the design of complex studies. A large share of the examined data came from short case series or case reports, reducing the level of evidence in our casuistry. Furthermore, our study, which is mainly focused on the outcomes of treatments comprising surgical removal of the disease as a pivotal step in both the diagnostic and therapeutic pathways, includes only cases treated surgically to reduce heterogeneity. However, this choice could have excluded cases treated without surgical interventions due to peculiar clinical conditions, palliative care, or experimental approaches. While their exclusion from our general population does not reasonably affect recurrence rates, their inclusion could have slightly increased the number of cases and changed the mean values of some assessed variables.

Despite these limitations, our review provides a structured synthesis of current evidence on musculoskeletal and peripheral nerve GCTs. By consolidating demographic, clinical, and outcome data, our findings contribute to a more nuanced understanding of these rare tumors. They may help clinicians recognize their presentation, select appropriate therapeutic strategies, and anticipate the oncological outcomes.

## 5. Conclusions

To date, the literature on musculoskeletal and peripheral nerve granular cell tumors remains limited and highly heterogeneous, largely consisting of isolated case reports and small case series. By systematically synthesizing available evidence, our review clarifies the spectrum of clinical presentation, anatomical distribution, and treatment strategies reported for these rare lesions. Overall, musculoskeletal GCTs should be considered in the differential diagnosis of slow-growing soft-tissue masses of the extremities, especially in female patients with firm localized swelling. Lesion size emerged as a potential predictor malignancy. Surgical excision remains the cornerstone of management, with low recurrence rates for both benign and malignant GCTs. The latter have a remarkable metastatic potential that significantly impacts patients’ life expectancy and overall survival after treatment.

Future directions in the management of granular cell tumors should focus on developing multicenter registries and conducting collaborative studies to overcome the limitations imposed by their rarity. Such efforts are needed to standardize prognostic stratification, refine surgical indications, and better define follow-up strategies, particularly for malignant variants.

## Figures and Tables

**Table 1:** A schematic resume of the data recorded from the single case series and (in the last row) a summary of all the case reports included in our review. N = Number of cases, Scapulr reg. = Scapular region, Abdom. wall = Abdominal wall, Recur. = Recurrence, F = Female, M = Male, B = Benign, A = Atypical, M = Malignant.

Article	N	Age	Sex	Tumor Type	Tumor Location	Tumor Size	Pain	Swelling	Recur.	MTS	FU
Elkousy et al. (2000) [15]	10	38 (15–59)	7 F 3 M	10 B	6 Thigh 3 Finger 1 Elbow	NA	40% (4)	100% (10)	0	0	27
Arai et al. (2010) [7]	5	40.2 (31–54)	4 F 1 M	3 B 1 A 1 M	3 Thigh 1 Leg 1 Gluteus	46 (30–70)	0	100% (5)	20% (1)	20% (1 Lung + nodes)	43
Blacksin et al. (2005) [16]	5	31.8 (13–56)	3 F 2 M	4 B 1 M	2 Thigh 1 Foot, 1 Shoulder, 1 Hand,	20.2 (7–42)	20% (1)	100% (5)	0	0	12
Singh et al. (2015) [8]	5	24 (13–40)	5 F	3 B 1 A 1 M	2 Thigh, 1 Scapular reg., 1 Arm, 1 Forearm	48 (20–80)	40% (2)	100% (5)	0	20% (1 Lungs)	14
Kudawara et al. (1999) [17]	3	45.7 (36–55)	3 F	3 B	1 Back, 1 Chest, 1 Scapular reg.,	33 (20–60)	0	100% (3)	0	0	33
Thacker et al. (2006) [18]	3	48 (35–64)	3 F	1 B 2 M	2 Gluteus 1 Arm	41.3 (24–50)	67% (2)	100% (3)	0	33% (1 Lungs)	12.3
Usui et al. (1982) [19]	2	16.5 (14–19)	1 F 1 M	2 B	2 Hand	6.5 (5–8)	50% (1)	100% (2)	50% (1)	0	27
Lee et al. (1994) [20]	2	11 (10–12)	2 F	2 B	1 Back, 1 Leg	16.5 (10–23)	100% (2)	100% (2)	0	0	19
Case Reports (1978–2025) [21,22,23,24,25,26,27,28,29,30,31,32,33,34,35,36,37,38,39,40,41,42,43,44,45,46,47,48,49,50,51,52]	32	43.1 (7–81)	2 F 10 M	19 B 13 M	9 Thigh 6 Hand 4 Abdom. wall 3 Forearm 2 Back 2 Neck 2 Shoulder 2 Arm Leg Knee Gluteus Pelvis	61.3 (5–200)	45% (15)	78.8% (26)	0	15.1% (2 Lung + Nodes, 1 Lung, 1 Lung + Liver, 1 Spine + Pelvis)	32

**Table 2:** The answers to all the queries of the JBI checklist for case series studies.

**Article**	**N**	**Q1**	**Q2**	**Q3**	**Q4**	**Q5**	**Q6**	**Q7**	**Q8**	**Q9**	**Q10**
Elkousy et al. (2000) [15]	10	Yes	Yes	Yes	Yes	Yes	Yes	Yes	Yes	Yes	NA
Arai et al. (2010) [7]	5	Yes	Yes	Yes	Yes	Yes	Yes	Yes	Yes	Yes	NA
Blacksin et al. (2005) [16]	5	Yes	Yes	Yes	Yes	Yes	Yes	Yes	Unclear	Yes	NA
Singh et al. (2015) [8]	5	Yes	Yes	Yes	Yes	Yes	Yes	Yes	Yes	Yes	NA
Kudawara et al. (1999) [17]	3	Yes	Unclear	Yes	Yes	Yes	Yes	Yes	Yes	Unclear	NA
Thacker et al. (2006) [18]	3	Yes	Yes	Yes	Yes	Yes	Yes	Yes	Yes	Yes	NA
Usui et al. (1982) [19]	2	Yes	Unclear	Yes	Yes	Yes	Yes	Yes	Unclear	Yes	NA
Lee et al. (1994) [20]	2	Yes	Unclear	Yes	Yes	Yes	Yes	Yes	Unclear	Yes	NA

**Table 3:** The answers to all the queries of the JBI checklist for case report articles.

**Authors**	**Q1**	**Q2**	**Q3**	**Q4**	**Q5**	**Q6**	**67**	**Q8**
Aaron et al. (1994) [24]	Yes	Yes	Yes	Yes	Unclear	No	No	Yes
Alnashwan et al. (2019) [42]	Yes	Yes	Yes	Yes	Yes	Yes	Yes	Yes
Aviles et al. (2014) [33]	Yes	Yes	Yes	Yes	Yes	Yes	Yes	Yes
Birişik et al. (2020) [36]	Yes	Yes	Yes	Yes	Yes	Yes	Yes	Yes
Chung et al. (2000) [27]	Yes	Yes	Yes	Yes	Yes	No	No	Yes
Costa et al. (2017) [35]	Yes	Yes	Yes	Yes	Yes	Yes	Yes	Yes
Deskoulidi et al. (2018) [45]	Yes	Yes	Yes	Yes	Yes	Yes	Yes	Yes
Donhuijsen et al. (1979) [22]	Yes	Unclear	Unclear	Yes	Yes	Unclear	Unclear	Yes
Enghardt & Jordan (1991) [40]	Yes	Yes	Yes	Yes	Yes	Unclear	Unclear	Yes
Gorelkin et al. (1978) [21]	Yes	Yes	Yes	Yes	Yes	Unclear	Unclear	Yes
Ha et al. (2011) [31]	Yes	Yes	Yes	Yes	Yes	Yes	Yes	Yes
Hobbs et al. (2022) [38]	Yes	Yes	Yes	Yes	Yes	Yes	Yes	Yes
Hurrell et al. (1995) [48]	Yes	Yes	Yes	Yes	Yes	No	No	Yes
Hyodo et al. (2001) [28]	Yes	Yes	Yes	Yes	Yes	Yes	Yes	Yes
Jardines et al. (1994) [52]	Yes	Yes	Yes	Yes	Yes	Yes	Yes	Yes
Kim et al. (2025) [47]	Yes	Yes	Yes	Yes	Yes	No	No	Yes
Korambayil et al. (2012) [32]	Yes	Yes	Yes	Yes	Yes	Yes	Yes	Yes
Maher et al. (1987) [23]	Yes	Yes	Yes	Yes	Yes	Yes	Yes	Yes
Nasit et al. (2013) [49]	Yes	Yes	Yes	Yes	Yes	Yes	Yes	Yes
Osanai et al. (2004) [29]	Yes	Yes	Yes	Yes	Yes	Yes	Yes	Yes
Porta et al. (2015) [46]	Yes	Yes	Yes	Yes	Yes	No	No	Yes
Raja et al. (2025) [39]	Yes	Yes	Yes	Yes	Yes	Yes	Yes	Yes
Saito et al. (2018) [51]	Yes	Yes	Yes	Yes	Yes	Yes	Yes	Yes
Salaouatchi et al. (2021) [37]	Yes	Yes	Yes	Yes	Yes	Yes	Yes	Yes
Saperstein et al. (1996) [25]	Yes	Yes	Yes	Yes	Yes	Yes	Yes	Yes
Shi et al. (2022) [44]	Yes	Yes	Yes	Yes	Yes	Yes	Yes	Yes
Slutsky J (2009) [41]	Yes	Yes	Yes	Yes	Yes	Yes	Yes	Yes
Torreggiani et al. (2001) [50]	Yes	Yes	Yes	Yes	Yes	Yes	Yes	Yes
Tsuchida et al. (1997) [26]	Yes	Yes	Yes	Yes	Yes	Yes	Yes	Yes
Wadhwa et al. (2014) [34]	Yes	Yes	Yes	Yes	Yes	No	No	Yes
Wen et al. (2022) [43]	Yes	Yes	Yes	Yes	Yes	No	No	Yes
Youssef et al. (2010) [30]	Yes	Yes	Yes	Yes	Yes	Yes	Yes	Yes

**Table 4:** A summary of treatments received by all cases included in our review, along with an overview of their surgical resection margins.

**Treatment**	**CASES**
**Therapeutic approaches**	
Surgery alone	63 (94.0%)
Surgery + Radiotherapy	2 (3.0%)
Surgery + Chemotherapy	1 (1.5%)
Surgery + Chemotherapy + Radiotherapy	1 (1.5%)
**Surgical Resection Margins**	
Margin unknown/not mentioned	39 (58.2%)
R0 or R1	26 (38.8%)
R2	2 (3.0%)

## Data Availability

This review is registered in Implasy register (Registration code: INPLASY202620034, DOI number:10.37766/inplasy2026.2.0034). The data that support the findings of this study are available from the corresponding author upon reasonable request.

## Supplementary Materials

The following supporting information can be downloaded at: https://www.mdpi.com/article/10.3390/diagnostics16060885/s1, PRISMA 2020 Checklists.

## Abbreviations

The following abbreviations are used in this manuscript:

AWD	Alive with disease
BRD7	Bromodomain-containing protein 7
CDF	Continuously disease free through their post-operative intercourse
DOC	Dead of other causes
DOD	Dead of disease
GCT	Granular cell tumor
GenAI	Generative artificial intelligence
GFRA2	GDNF Family Receptor Alpha 2
JBI	Joanna Briggs Institute
MeSH	Medical Subject Headings
MRI	Magnetic Resonance Imaging
PRISMA	Preferred Reporting Items for Systematic Reviews and Meta-Analyses
TP53	Tumor Protein P53
WHO	World Health Organization
